# Mortality in relation to diabetes remission in Swedish Obese Subjects – a prospective cohort study

**DOI:** 10.1097/JS9.0000000000001807

**Published:** 2024-06-19

**Authors:** Lena M.S. Carlsson, Björn Carlsson, Peter Jacobson, Cecilia Karlsson, Johanna C. Andersson-Assarsson, Felipe M. Kristensson, Sofie Ahlin, Per-Arne Svensson, Magdalena Taube, Ingmar Näslund, Kristjan Karason, Markku Peltonen, Kajsa Sjöholm

**Affiliations:** a Institute of Medicine, Sahlgrenska Academy, University of Gothenburg, Gothenburg; b Research and Early Development, Cardiovascular, Renal and Metabolism (CVRM), BioPharmaceuticals R&D, AstraZeneca, Gothenburg; c Late-Stage Development, Cardiovascular, Renal and Metabolism (CVRM), BioPharmaceuticals R&D, AstraZeneca, Gothenburg; d Department of Surgery, Region Västra Götaland, Sahlgrenska University Hospital/Östra, Gothenburg; e Institute of Health and Care Sciences, Sahlgrenska Academy, University of Gothenburg, Gothenburg; f Department of Clinical Physiology, Region Västra Götaland, NU Hospital Group, Trollhättan; g Department of Surgery, Faculty of Medicine and Health, Örebro University, Örebro, Sweden; h Finnish Institute for Health and Welfare, Helsinki, Finland

**Keywords:** cause-specific mortality, diabetes remission, life expectancy, obesity, type 2 diabetes

## Abstract

**Background:**

People with obesity and type 2 diabetes (T2D) have reduced life expectancy, partly explained by increased risk of cardiovascular diseases and cancer. Here, we examined whether 2-year diabetes remission after bariatric surgery or usual care is associated with long-term mortality.

**Materials and methods:**

This report includes 586 participants with obesity and concomitant T2D from the prospective Swedish Obese Subjects (SOS) cohort study; 338 underwent bariatric surgery and 248 received usual obesity care. At inclusion, age was 37–60 years and BMI ≥34 kg/m^2^ in men and ≥38 kg/m^2^ in women. Median follow-up was 26.2 years (interquartile range 22.7–28.7). Diabetes status was determined using self-reported data on diabetes medication and in-study measures of blood glucose and HbA1c. The study was cross-linked to Swedish national registers for data on morbidity, death, and emigration.

**Results:**

Overall, 284 participants, 71.9% of surgery and 16.5% of usual care patients were in remission at the 2-year examination. During follow-up, mortality rates were 16.6 deaths per 1000 person-years (95% CI: 13.7–20.1) in the remission subgroup and 26.0 deaths per 1000 person-years (95% CI:22.2–30.4) in the non-remission subgroup (adjusted hazard ratio (HR_adj_)=0.71, 95% CI:0.54–0.95, *P*=0.019). The adjusted median life expectancy in the remission subgroup was 2.5 years (95% CI:0.3–4.7) longer than in the non-remission subgroup. Specifically, remission was associated with decreased cardiovascular mortality (sub-HR_adj_=0.54, 95% CI: 0.35–0.85, *P*=0.008), but no detectable association with cancer mortality was found (sub-HR_adj_=1.06, 95% CI:0.60–1.86), *P*=0.841).

**Conclusion:**

In this post-hoc analysis of data from the SOS study, patients who achieved short-term diabetes remission had increased life expectancy and decreased cardiovascular death over up to 32 years of follow-up. Future studies should confirm these findings.

## Introduction

Highlights

- T2D is a progressive disease, but limited knowledge exists about the association between short-term diabetes remission and longevity.
- Patients who achieved diabetes remission at the 2-year follow-up had significantly reduced mortality rates over 32 years compared to those not achieving remission.
- Lower mortality was mainly due to a decreased risk of cardiovascular death.
- In this post-hoc analysis of data from the SOS study, patients who achieved short-term diabetes remission had increased life expectancy and especially decreased risk of cardiovascular death.

Cardiovascular disease (CVD) and cancer are the two most common causes of death in people with obesity, and those who suffer from both obesity and type 2 diabetes (T2D) have the highest risk for serious comorbidities and a shortened life expectancy^1,2^. Bariatric (metabolic) surgery has positive effects on cardiovascular and cancer-related morbidity and mortality^3–8^, thereby partly reversing the decreased life expectancy for patients with obesity and concomitant T2D^9–11^.

Randomized controlled trials have demonstrated that surgery-induced weight loss in patients with obesity and T2D is very effective for improved metabolic control^12^. Moreover, intentional weight loss in people with obesity and T2D, whether induced by lifestyle modification or bariatric surgery, is associated with diabetes remission in a large proportion of individuals with T2D^5,13–15^, although many relapse over time^5,16^. Insulin production is already impaired during the prediabetic phase and is often reduced by up to 80% at the time of T2D diagnosis, reflecting the progressive nature of T2D^17^. In people with obesity and concomitant T2D, the likelihood of remission^18^ depends on the duration of T2D and the degree of weight loss after surgical or dietary interventions^19–24^.

Whether remission is associated with long-term mortality rates or specific causes of death in individuals with obesity and T2D is largely unknown. Thus, the aim of this study was to investigate whether remission status after bariatric surgery or usual care is associated with long-term mortality by comparing death and comorbidity rates in relation to 2-year diabetes remission in participants with T2D at baseline in the Swedish Obese Subjects (SOS) study.

## Methods

### Study design

**Figure 1:**
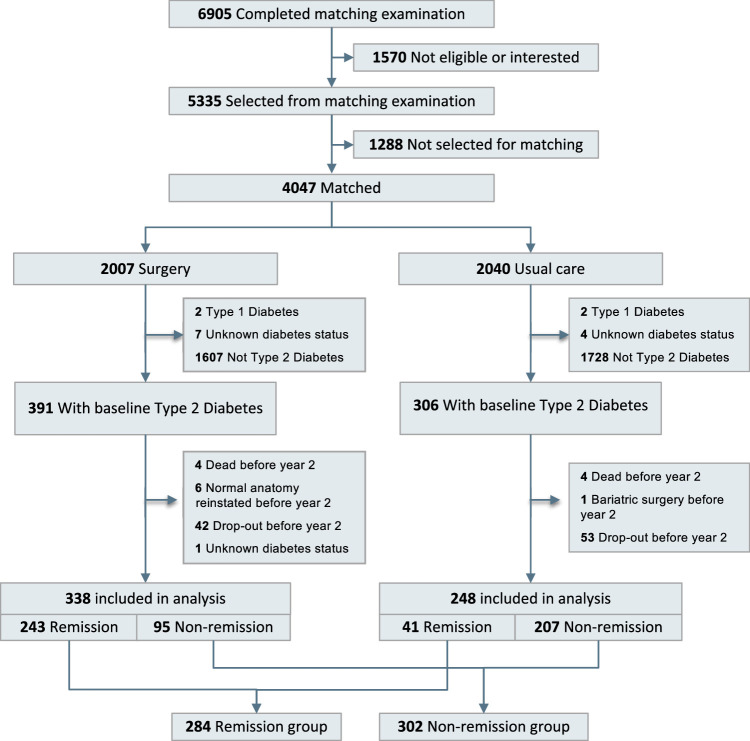
Flowchart for the Swedish Obese Subjects (SOS) study describing stratification into remission and non-remission subgroups for mortality analyses.

The SOS study was designed to compare the effects of bariatric surgery and usual care in participants with obesity^2,5,25^, and was conducted at 25 surgical departments and 480 primary healthcare centers in Sweden. As a result of recruitment campaigns in the mass media and at primary healthcare centers, 6905 persons completed a matching examination and 5335 of them were eligible for inclusion in the SOS study. Participants were recruited between 1 September 1987 and 31 January 2001. The seven regional ethics review boards in Sweden approved the study protocol, and written or oral informed consent was obtained from all participants. The per-protocol surgery group constituted *n*=2007 individuals electing surgery, and a contemporaneously matched control group receiving usual obesity care (*n*=2040) was created based on 18 matching variables (Supplementary Appendix, Supplemental Digital Content 1, http://links.lww.com/JS9/C807). Surgery patients underwent vertical banded gastroplasty, gastric banding, or gastric bypass, and the usual care group received usual obesity care. The inclusion criteria were age 37–60 years and body mass index (BMI) of 34 kg/m^2^ or more for men and 38 kg/m^2^ or more for women, corresponding to a doubling in the rate of death in each sex^26^. The exclusion criteria were identical in surgery patients and the usual care group and selected to enroll patients that could undergo surgery (Supplementary Appendix, Supplemental Digital Content 1, http://links.lww.com/JS9/C807). Self-reported questionnaire data, as well as urine and fasting blood samples, were obtained at matching, baseline, and at the 2-year and 10-year follow-up examinations. Self-reported data on diabetes duration, diabetes medication, and CVD or cancer-related morbidity before baseline were obtained from SOS questionnaires. In this report, we examine the association between 2-year diabetes remission and mortality during follow-up in participants with T2D at baseline. A study flow chart is provided in Figure 1. This trial is registered at https://clinicaltrials.gov/. The work has been reported in line with the STROCSS criteria^27^ (Supplemental Digital Content 2, http://links.lww.com/JS9/C808).

### Diabetes-related definitions

Diabetes status was determined at baseline and at the 2-year and 10-year follow-up examinations for the current analyses, and it was based on in-study measures of blood glucose and HbA1c and self-reported data on diabetes medication. Baseline diabetes was defined as the use of diabetes medication, a single determination of fasting blood glucose level of ≥6.1 mmol/l (corresponding to fasting plasma glucose of ≥7.0 mmol/l) or HbA1c ≥48 mmol/mol^28^. In patients with diabetes onset before the age of 35, we excluded those that were positive for glutamate decarboxylase antibodies or islet cell antibodies or with C-peptide values below the detection limit to rule out type 1 diabetes and latent autoimmune diabetes in adults (LADA). This resulted in the exclusion of four patients, two surgery cases, and two patients who were treated with usual care. T2D remission was defined as HbA1c <48 mmol/mol or fasting blood glucose <6.1 mmol/l (plasma glucose <7.0 mmol/l) without receipt of diabetes medication. Remission was assessed at the 2-year examination and at the 10-year examination to identify individuals with diabetes relapse^23^.

Verification of T2D diagnosis by repeated measures was not standard routine in the 1980s when the SOS study was started. A sensitivity analysis was therefore performed where diabetes identified with a single measure of blood glucose (≥6.1 mmol/l) or HbA1c (≥48 mmol/mol) was confirmed by one of the following: self-report of either T2D medication or dietary treatment of T2D, self-report of a diabetes-related complication, HbA1c level of ≥48 mmol/mol or blood glucose of ≥6.1 mmol/l at the matching examination or elevated urinary glucose levels at the matching and/or baseline examination.

### Overall mortality, causes of death, and cause-specific morbidity

Information on all deaths until 31 December 2020, was obtained by cross-checking the SOS database against the Swedish Population and Address Register (SPAR). Information on the official cause of death was obtained from the Swedish Cause of Death Register. Since the official cause of death reflects the underlying preventable cause (e.g. obesity) in the chain of events leading to death, relevant case sheets and autopsy reports were assessed independently by two authors to determine the direct cause of death^2^. The study-determined direct cause of death was used if the official and direct cause of death differed. Data on cancer incidence were obtained from the Swedish National Cancer Registry^4^, which provides close to complete coverage for all malignant tumors. Macrovascular and microvascular events were captured from the National Patient Register using the International Classification of Diseases and intervention codes listed in Supplementary Table S1 (Supplemental Digital Content 1, http://links.lww.com/JS9/C807)^5,25^.

### Statistical methods

Data are presented as mean values with standard deviations or as numbers with percentages. Baseline and 2-year characteristics between remission and non-remission groups were compared with *t* tests for continuous variables and Fisher exact test for dichotomous variables. BMI and body weight changes in remission and non-remission groups were analyzed with multilevel mixed-effects regression models. The observations were considered nested within the individuals, and the confidence intervals were thus calculated, controlling for repeated measurements. Kaplan–Meier estimates of survival functions were calculated for remission and non-remission groups, and the cumulative incidence is presented as one minus the Kaplan–Meier estimate. The associations between 2-year diabetes remission, overall mortality, and cause-specific morbidity were estimated with Kaplan–Meier estimates and Cox proportional hazard regression models, and the results are presented as hazard ratios (HR) with 95% confidence intervals (CI). Basic adjustments (model 1) accounted for preselected risk factors (age, sex, BMI, and smoking at baseline, and year of inclusion in the study). In subsequent analyses, we also adjusted for diabetes duration (model 2) and previous CVD, hypertension, and total cholesterol (model 3, full adjustment denoted HR_adj_). We used the Gompertz proportional hazards regression model to compare life expectancy between remission and non-remission groups^2^. All survival analyses were conditioned on persons surviving and participating in the 2-year health examination in order to create the remission/non-remission groups. Consequently, those who died or did not participate in the 2-year health examination for any reason were excluded from the analyses.

In analyses on total mortality, participants who were lost to follow-up after the initial 2 years or were alive on the 31st of December 2020 were treated as censored observations. Persons in the usual care group who received bariatric surgery and persons in the surgery group who had their anatomy restored during the post-2-year follow-up were censored at the operation date. A sensitivity analysis based on multiple imputations of missing remission data was performed to cover those 16.5% of the study population who were excluded from the main analyses due to missing data. In addition, a sensitivity analysis, only including individuals in whom an additional in-study measure validated the T2D diagnosis, was conducted. Analyses of cause-specific mortality were conducted with the competing-risks regression models suggested by Fine and Gray^29^, in which deaths for other reasons were treated as competing events. The association between 2-year remission and cause-specific mortality was evaluated in a primary unadjusted analysis with a single covariate for diabetes status (remission or non-remission) and is expressed as a sub-hazard ratio (sub-HR) with 95% CI. Patients were included in the analysis according to the intervention they received (i.e. as treated). All statistical tests were two-sided, and *P* values of less than 0.05 were considered to indicate statistical significance. Stata software, version 15.1 (StataCorp), was used for all analyses.

## Results

### Participants

In the current report, we only included SOS study participants (*n*=586) with baseline type 2 diabetes who were alive at the 2-year follow-up and for whom remission status at 2 years was known (Fig. 1). Baseline characteristics stratified by diabetes status at the 2-year examination, for the full cohort (i.e. all participants with baseline T2D in both treatment groups) and separate for 338 surgery patients and 248 patients in the usual care group, are shown in Table 1. On average, patients who were in remission at 2 years had higher BMI, lower blood glucose and glycated hemoglobin, and shorter diabetes duration at baseline compared to patients who did not achieve remission. A total of 284 patients were in remission at 2 years; and 243 (85.6%) of them had undergone bariatric surgery, and 41 (14.4%) had been given usual care. The diabetes relapse rate between years 2 and 10 was 54.1% in surgery patients and 70.4% in the usual care group (*P*=0.146). The median follow-up time was 26.2 years (interquartile range 22.7–28.7 years), and the maximum follow-up was 32.2 years).

**Table 1:** Baseline characteristics for the full cohort, and separate for surgery patients and usual care controls, stratified by 2-year remission status.

	Full cohort			Surgery^a^			Control		
	Remission	Non-remission	*P* b	Remission	Non-remission	*P* b	Remission	Non-remission	*P* b
*N*	284	302		243	95		41	207	
Age, years	48.9±6.0	50.2±6.2	0.010	48.7±5.9	49.1±5.9	0.656	50.0±6.2	50.8±6.3	0.495
Male sex, *N* (%)	110 (38.7)	119 (39.4)	0.933^c^	91 (37.4)	39 (41.1)	0.537^c^	19 (46.3)	80 (38.6)	0.386^c^
Body weight, kg	124.2±19.7	117.1±16.8	<0.001	124.6±20.1	119.5±15.8	0.014	121.3±17.4	116.0±17.1	0.079
Waist circumference, cm	128.9±12.4	124.0±10.2	<0.001	129.3±12.7	127.5±9.7	0.155	126.6±10.3	122.5±10.0	0.022
BMI, kg/m^2^	42.7±5.0	40.2±4.4	<0.001	42.9±5.0	41.0±3.7	<0.001	41.5±4.3	39.8±4.6	0.033
Blood glucose, mmol/l	7.4±2.3	8.8±2.9	<0.001	7.5±2.3	10.0±2.8	<0.001	6.4±1.7	8.3±2.8	<0.001
Glycated hemoglobin, %	7.3±1.4	8.1±1.5	<0.001	7.4±1.4	8.7±1.5	<0.001	6.5±0.8	7.9±1.4	<0.001
Glycated hemoglobin, mmol/mol	56.0 ±14.9	65.4±16.0	<0.001	57.5±15.2	71.7±15.9	<0.001	47.4±8.4	62.5±15.3	<0.001
Serum insulin, pmol/l	169.7±93.4	151.1±93.6	0.017	168.8±79.6	158.6±101.7	0.384	174.8±152.5	147.6±89.7	0.276
HOMA-IR	10.6±7.6	11.0±7.8	0.552	10.6±6.5	13.1±9.3	0.019	10.2±12.1	10.0±6.8	0.904
Diabetes duration, years	0.8±1.9	4.4±5.3	<0.001	1.0±2.0	6.7±6.3	<0.001	0.2±0.9	3.4±4.5	<0.001
Total cholesterol, mmol/l	5.9±1.3	5.7±1.1	0.024	6.0±1.3	5.9±1.2	0.277	5.5±1.2	5.6±1.1	0.590
HDL cholesterol, mmol/l	1.2±0.3	1.2±0.3	0.905	1.2±0.3	1.3±0.3	0.545	1.2±0.3	1.2±0.3	0.961
LDL cholesterol, mmol/l	3.4±1.0	3.3±1.0	0.044	3.4±1.0	3.3±1.1	0.246	3.4±1.0	3.2±1.0	0.327
Triglycerides, mmol/l	2.8±2.4	2.7±1.8	0.609	3.0±2.5	2.8±1.4	0.542	2.0±0.9	2.7±2.0	0.001
Non-HDL cholesterol, mmol/l	4.6±1.2	4.4±1.2	0.211	4.6±1.2	4.6±1.2	0.763	4.3±1.2	4.4±1.2	0.825
Systolic blood pressure, mmHg	148.9±19.2	147.0±19.0	0.252	149.9±19.2	152.5±18.8	0.272	142.4±18.6	144.6±18.6	0.509
Diastolic blood pressure, mmHg	91.3±11.6	88.6±11.3	0.004	91.8±11.8	91.5±10.5	0.845	88.2±9.7	87.2±11.3	0.559
SCORE 10-year risk^d^	2.4±2.9	2.5±3.0	0.649	2.3±2.7	2.4±2.7	0.611	3.0±3.7	2.5±3.2	0.429
Smoking daily, *N* (%)	65 (22.9)	63 (21.0)	0.617^c^	56 (23.0)	19 (20.2)	0.662^c^	9 (22.0)	44 (21.4)	1.000^c^
Alcohol, g/day	5.2±7.7	5.0±7.4	0.703	4.8±7.3	5.0±7.3	0.824	7.6±9.8	4.9±7.5	0.137
Previous cardiovascular disease, *N* (%)^e^	8 (2.8)	22 (7.3)	0.015^c^	7 (2.9)	12 (12.6)	0.001^c^	1 (2.4)	10 (4.8)	0.697^c^
Previous cancer, *N* (%)^e^	5 (1.8)	7 (2.3)	0.773^c^	4 (1.6)	2 (2.1)	0.675^c^	1 (2.4)	5 (2.4)	1.000^c^

Data are mean±SD or *n* (%).

a Surgical procedure in remission/non-remission groups: vertical banded gastroplasty (*N*=148/*N*=68), nonadjustable or adjustable banding (*N*=47/*N*=17), or gastric bypass (*N*=48/*N*=10).

b Difference between remission and non-remission groups.

c Fischer’s exact test for categorical variables.

d European SCORE (Systematic Coronary Risk Evaluation) model (Eur Heart J 2003; 24: 987–1003).

e Self-reported data from the SOS questionnaire.

### Overall mortality and life expectancy

**Figure 2:**
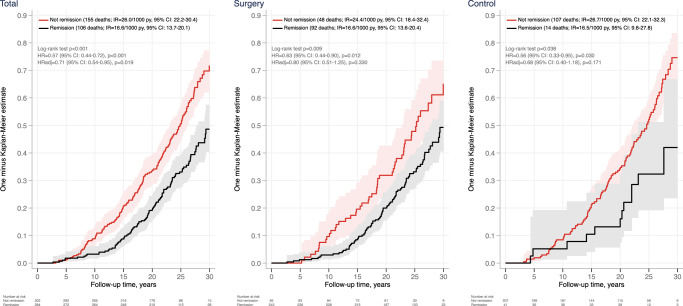
Cumulative mortality in the full cohort (left panel), surgery (middle panel), and usual care (right panel) groups of the SOS study by diabetes remission at 2 years. Per-protocol analysis adjusted for BMI, age, sex, smoking, inclusion year, diabetes duration, previous cardiovascular disease (CVD), hypertension, and total cholesterol. IR/1000, the incidence rate per 1000 person-years.

During follow-up, there was a total of 261 deaths, and Figure 2 shows cumulative overall mortality stratified by T2D remission at 2 years. In the full cohort, there were 106 (incidence rate (IR)=16.6/1000 person-years, 95% CI: 13.7–20.1) and 155 (IR=26.0 (22.2–30.4)) deaths in the subgroups with and without diabetes remission at year 2, respectively. Diabetes remission at year 2 was associated with reduced overall mortality (crude HR=0.57 (0.44–0.72), *P*<0.001) (Fig. 2, left panel). Subsequently, we performed a stepwise inclusion of baseline risk factors into the regression model to assess the influence of different variables on mortality risk estimates. The point estimate was similar after adjustment for age, sex, BMI, smoking, and year of inclusion (Model 1; HR=0.59 (0.46–0.77), *P*<0.001) (Table 2). However, the strength of the association was attenuated when additional risk factors were added. In model 2, which also included diabetes duration as a covariate, the resulting HR was 0.72 (0.54–0.95, *P*=0.021), and after full adjustment, including hypertension, total cholesterol, and previous CVD (model 3), the resulting HR_adj_ was 0.71 (0.54–0.95, *P*=0.019) (Table 2).

**Table 2:** Association between 2-year diabetes remission and mortality.

	Model 1		Model 2		Model 3	
	HR	95% CI	HR	95% CI	HR_adj_	95% CI
Full cohort (*n*=284 remission; 302 non-remission)	0.59	0.46–0.77	0.72	0.54–0.95	0.71	0.54–0.95
Surgery (*n*=243 remission; *n*=95 non-remission)	0.57	0.39–0.83	0.79	0.51–1.24	0.80	0.51–1.25
Usual care (*n*=41 remission; *n*=207 non-remission)	0.53	0.32–0.90	0.67	0.39–1.15	0.68	0.40–1.18

Hazard ratios (HR) and 95% confidence intervals (CI) are from Cox proportional hazards models. Model 1 is adjusted for age, sex, BMI and smoking at baseline, and year of inclusion in the study. Model 2 is model 1 + diabetes duration. Model 3 (full adjustment) is model 2 + previous CVD, hypertension, and total cholesterol.

The median life expectancy was 4.8 years (95% CI 2.6–6.9) longer in the remission subgroup than in the non-remission subgroup (*P*<0.001) (adjusted difference, 2.5 years (95% CI 0.3–4.7); *P*<0.023) (Supplementary Fig. S1, Supplemental Digital Content 1, http://links.lww.com/JS9/C807). Moreover, in a sensitivity analysis with multiple imputations of the missing 2-year remission data, the difference in mortality rate among those who achieved remission compared to those who did not remained statistically significant (HR_adj_=0.72 (0.55–0.94), *P*=0.015) (Supplementary Table S3, Supplemental Digital Content 1, http://links.lww.com/JS9/C807).

When the cohort was stratified by the intervention (Fig. 2, middle and right panels), diabetes remission at year 2 was associated with significantly reduced mortality in the surgery (HR=0.57 (0.39–0.83), *P*=0.003) and usual care (HR=0.53 (0.32–0.90), *P*=0.018) groups after basic adjustments (Table 2, model 1). The associations were no longer significant after the addition of diabetes duration as a covariate, although risk estimates were similar to those in the full cohort regardless of the model (Table 2). However, it is important to acknowledge that these stratified analyses are subject to a higher degree of uncertainty due to the smaller sample sizes, the limited number of patients achieving 2-year remission in the usual care group, and the small number of patients who did not achieve remission at 2 years in the surgery group.

The SOS study was initiated before repeated measurements were routinely used for the diagnosis of type 2 diabetes. To verify our results, we therefore performed a sensitivity analysis including only individuals where an additional in-study measure validated the T2D diagnosis (*n*=524) (Supplementary Fig. S2, Supplemental Digital Content 1, http://links.lww.com/JS9/C807). This analysis, based on a total of 237 deaths, confirmed an association between 2-year remission and reduced overall mortality in the full cohort (HR_adj_=0.69 (0.51–0.94), *P*=0.018).

### Cause-specific mortality and morbidity

Mortality in the full cohort included 106 deaths from cardiovascular diseases, 63 from malignancies and 92 from other causes. Diabetes remission at year 2 was associated with reduced cardiovascular mortality (sub-HR_adj_=0.54 (0.35–0.85), *P*=0.008) (Table 3), and more detailed analysis showed that remission at year 2 was specifically associated with reduced risk of death due to myocardial infarction (sub-HR_adj_=0.43 (0.19–0.97), *P*=0.042), whereas no difference was detected for other cardiac outcomes or stroke (Supplementary Table S4, Supplemental Digital Content 1, http://links.lww.com/JS9/C807). In contrast, cancer-related mortality (sub-HR_adj_=1.06 (0.60–1.86), *P*=0.841), and mortality due to various other causes (sub-HR_adj_=0.95 (0.59–1.54), *P*=0.835) were similar in the groups with and without diabetes remission at year 2 (Table 3). However, infection-related mortality was less common in the remission group (sub-HR_adj_=0.35 (0.14–0.88) *P*=0.026) (Supplementary Table S4, Supplemental Digital Content 1, http://links.lww.com/JS9/C807).

**Table 3:** Cause-specific mortality in the full cohort, stratified by 2-year diabetes remission.

	Remission (*N*=284)	IR/1000 person-years	Non-remission (*N*=302)	IR/1000 person-years	Adj. sub-HR (95% CI)^a^	Adj. *P* for sub-HR^a^
Cardiovascular	40	6.3 (95% CI: 4.6–8.6)	66	11.1 (95% CI: 8.7–14.1)	0.54 (0.35–0.85)	0.008
Cardiac	33		60			
MI	9		26			
Heart failure	5		10			
Sudden death	18		24			
Other cardiac	1		0			
Stroke	6		6			
Other cardiovascular^b^	1		0			
Malignancy	29	4.5 (95% CI: 3.2–6.5)	34	5.7 (95% CI: 4.1–8.0)	1.06 (0.60–1.86)	0.841
Other	37	5.8 (95% CI: 4.2–8.0)	55	9.2 (95% CI: 7.1–12.0)	0.95 (0.59–1.54)	0.835
Complications after bariatric surgery	1		1			
Complications after other surgery	2		1			
Infection	7		23			
Neurological disease	5		2			
Kidney disease	0		8			
Liver disease	5		1			
Gastrointestinal disease	1		0			
Lung disease	2		1			
Thromboembolic disease^c^	1		2			
Causes other than disease^d^	6		5			
Other or multiple conditions	4		11			
Unknown cause of death	3		0			

Sub-hazard ratios (sub-HR) with 95% confidence intervals (CI) from Fine and Gray competing risk regression models (per-protocol analysis).

a Adjusted for sex, age, BMI, smoking, year of inclusion, diabetes duration, previous CVD, hypertension, and total cholesterol.

b Aortic aneurysm.

c Pulmonary embolia or embolism.

d This includes alcohol abuse, trauma, accident, and suicide.

**Figure 3:**
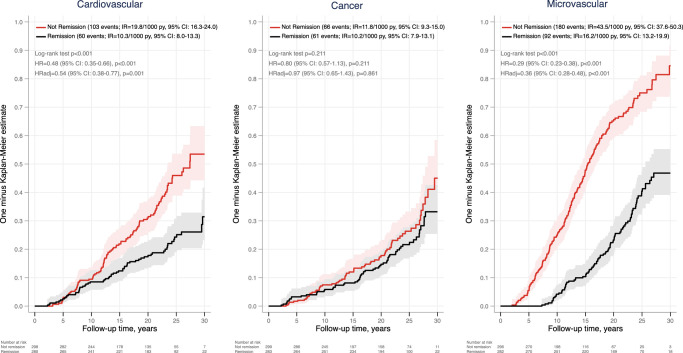
Cumulative incidence of cardiovascular disease (CVD), malignancy, and microvascular events in the full cohort by diabetes remission at 2 years. Per-protocol analysis adjusted for BMI, age, sex, smoking, inclusion year, diabetes duration, previous CVD, hypertension, and total cholesterol. IR/1000, the incidence rate per 1000 person-years.

In a subsequent analysis, we also tested the association between 2-year remission and cause-specific morbidity. In line with the mortality results, 2-year remission was associated with decreased risk of CVD (HR_adj_=0.54 (0.38–0.77), *P*=0.001) but not with incident cancer (Fig. 3, left and middle panels). Moreover, 2-year remission was associated with a reduced risk of incident microvascular complications (HR_adj_=0.36 (0.28–0.48), *P*<0.001) (Fig. 3, right panel).

### BMI and risk factor changes over 2 years

BMI and body weight are presented in Supplementary Figure S3 (Supplemental Digital Content 1, http://links.lww.com/JS9/C807). In the full cohort, mean (SD) BMI at baseline was 42.7 (5.0) in remission patients and 40.2 (4.4) in non-remission patients (Supplementary Table S2, Supplemental Digital Content 1, http://links.lww.com/JS9/C807). Apart from LDL cholesterol and total cholesterol, other risk factor improvements were more pronounced in remission patients (Supplementary Table S2, Supplemental Digital Content 1, http://links.lww.com/JS9/C807).

When stratified by intervention, mean (SD) 2-year weight changes in remission and non-remission patients from the surgery group amounted to −30.9 (15.5) and −18.5 (10.7) kg, respectively (*P*<0.001) which may be compared to −6.8 (9.4) and −2.5 (6.7) kg, respectively, in remission and non-remission patients from the usual care group (*P*=0.007) (Table 4). In general, the magnitude of risk factor changes at year 2 was greater in surgery patients. However, regardless of intervention, participants in remission displayed significantly larger improvements in measures of anthropometry and insulin levels (Table 4).

**Table 4:** Baseline characteristics and 2-year changes in surgery patients and usual care controls by 2-year remission status.

	Surgery			Control		
	Remission (*n*=243)	Non-remission (*n*=95)	*P*	Remission (*n*=41)	Non-remission (*n*=207)	*P*
Body weight, kg						
Baseline	124.6±20.1	119.5±15.8	0.014	121.3±17.4	116.0±17.1	0.079
2 years	93.7±17.1	101.0±17.5	0.001	114.5±17.8	113.6±17.4	0.760
Absolute change	−30.9±15.5	−18.5±10.7	<0.001	−6.8±9.4	−2.5±6.7	0.007
Waist circumference, cm						
Baseline	129.3±12.7	127.5±9.7	0.155	126.6±10.3	122.5±10.0	0.022
2 years	105.9±13.4	113.3±13.5	<0.001	120.9±11.2	121.3±10.4	0.854
Absolute change	−23.4±12.7	−14.2±10.2	<0.001	−5.7±7.5	−1.2±6.2	0.001
Body mass index, kg/m^2^						
Baseline	42.9±5.0	41.0±3.7	<0.001	41.5±4.3	39.8±4.6	0.033
2 years	32.3±4.8	34.6±4.9	<0.001	39.2±4.7	39.0±4.7	0.841
Absolute change	−10.6±5.2	−6.4±3.6	<0.001	−2.3±3.2	−0.8±2.3	0.008
Blood glucose, mmol/l						
Baseline	7.5±2.3	10.0±2.8	<0.001	6.4±1.7	8.3±2.8	<0.001
2 years	4.4±0.6	7.0±2.5	<0.001	4.6±0.6	8.7±2.9	<0.001
Absolute change	−3.2±2.4	−2.9±3.6	0.510	−1.9±1.7	0.4±2.8	<0.000
HbA1c, mmol/mol						
Baseline	57.5±15.2	71.7±15.9	<0.001	47.4±8.4	62.5±15.3	<0.001
2 years	39.1±4.1	57.0±14.6	<0.001	42.1±4.1	69.7±17.4	<0.001
Absolute change	−18.4±14.9	−15.3±18.0	0.156	−5.0±7.8	7.3±16.0	<0.000
Serum insulin, pmol/l						
Baseline	168.8±79.6	158.6±101.7	0.384	174.8±152.5	147.6±89.7	0.276
2 years	68.4±38.6	97.9±57.1	<0.001	101.0±44.3	139.1±92.3	<0.001
Absolute change	−100.4±77.7	−62.8±90.6	0.001	−73.8±139.6	−8.2±84.8	0.006
HOMA-IR						
Baseline	10.6±6.5	13.1±9.3	0.019	10.2±12.1	10.0±6.8	0.904
2 years	2.5±1.6	6.1±5.6	<0.001	3.9±1.8	9.9±6.6	<0.001
Absolute change	−8.1±6.4	−7.1±9.5	0.358	−6.3±11.6	−0.1±6.7	0.002
Total cholesterol, mmol/l						
Baseline	6.0±1.3	5.9±1.2	0.277	5.5±1.2	5.6±1.1	0.590
2 years	5.7±1.3	5.5±1.1	0.127	5.5±1.1	5.6±1.2	0.700
Absolute change	−0.3±1.0	−0.3±1.0	0.717	−0.0±0.9	−0.1±0.8	0.777
HDL cholesterol, mmol/l						
Baseline	1.2±0.3	1.3±0.3	0.545	1.2±0.3	1.2±0.3	0.961
2 years	1.5±0.4	1.4±0.3	0.149	1.3±0.4	1.3±0.3	0.172
Absolute change	0.3±0.3	0.2±0.3	0.005	0.1±0.2	0.0±0.2	0.134
Non-HDL cholesterol, mmol/l						
Baseline	4.6±1.2	4.6±1.2	0.763	4.3±1.2	4.4±1.2	0.825
2 years	4.2±1.3	4.0±1.1	0.230	4.2±1.1	4.2±1.1	0.755
Absolute change	−0.4±0.9	−0.5±1.0	0.580	−0.2±0.9	−0.1±0.7	0.579
LDL cholesterol, mmol/l						
Baseline	3.4±1.0	3.3±1.1	0.246	3.4±1.0	3.2±1.0	0.327
2 years	3.5±1.1	3.1±0.9	0.004	3.3±0.9	3.1±1.0	0.269
Absolute change	0.0±0.8	−0.1±1.0	0.134	−0.1±0.9	−0.1±0.6	0.889
Triglycerides, mmol/l						
Baseline	3.0±2.5	2.8±1.4	0.542	2.0±0.9	2.7±2.0	0.001
2 years	1.7±1.0	2.1±1.2	0.003	1.8±1.0	2.6±1.6	<0.001
Absolute change	−1.3±2.1	−0.7±1.3	0.004	−0.2±0.7	−0.1±1.3	0.399
Systolic blood pressure, mmHg						
Baseline	149.9±19.2	152.5±18.8	0.272	142.4±18.6	144.6±18.6	0.509
2 years	138.4±21.4	143.9±23.0	0.049	137.8±16.5	142.6±17.8	0.092
Absolute change	−11.7±20.2	−8.6±23.5	0.258	−4.7±16.6	−1.9±16.5	0.331
Diastolic blood pressure, mmHg						
Baseline	91.8±11.8	91.5±10.5	0.845	88.2±9.7	87.2±11.3	0.559
2 years	83.8±11.3	85.9±11.7	0.134	84.8±9.4	85.2±9.4	0.809
Absolute change	−8.0±11.3	−5.7±11.9	0.097	−3.5±11.3	−2.1±10.2	0.468

Data are mean±SD.

## Discussion

This study demonstrates that short-term diabetes remission is associated with reduced long-term mortality and increased life expectancy in the SOS cohort. Although results failed to reach significance in fully adjusted intervention-stratified analyses, the hazard ratio for overall mortality in those with diabetes remission was similar in patients treated with surgery and patients who received usual care.

Diabetes remission after bariatric surgery has been associated with decreased mortality compared with patients who did not achieve remission in retrospective studies with up to 5.8 years of median follow-up^30,31^. However, since the risk of diabetes relapse increases with time, it is possible that the positive effect of remission on survival is transient. Thus, whether short-term (2-year) diabetes remission is associated with mortality over several decades in patients with obesity and concomitant T2D has previously not been tested. Here we demonstrate an association between remission at the 2-year follow-up and reduced mortality rate (by 29%) over up to 30 years in the full cohort. The lower mortality corresponded with a 2.5-year longer adjusted median life expectancy in patients who were in remission at 2 years. Moreover, risk estimates in surgery patients and usual care controls were in the same range as for the full cohort, although the association was not significant after full adjustment in either treatment group. It is possible that this is partly due to low power as there was only a small number of patients in remission in the usual care group and a small number of non-remission patients in the surgery group.

It is well established that better treatment efficacy can be expected in patients with a recently diagnosed T2D and that weight loss improves glycemic control in patients with obesity and concomitant T2D^5,16,20,22,23,25^. In agreement, both surgery and usual care patients who were able to achieve remission had shorter diabetes duration and displayed a larger degree of weight loss compared to non-remission subgroups. Patients who were able to achieve 2-year remission after usual obesity care displayed a more transient and modest weight loss than those treated by surgery. However, it needs to be acknowledged that in addition to weight reduction, other demographic or lifestyle factors (e.g. dietary habits, physical activity) also may play a role in this context. Most patients who achieved 2-year remission had been treated by bariatric surgery and lost a substantial amount of weight. We and others have previously demonstrated that the degree of weight loss is associated with long-term glycemic control^5,21,32^, and a recent study demonstrated that surgery patients were more likely to achieve remission with increasing weight loss, although remission rates stabilized for weight loss degrees over 20%^20^. The degree of weight loss has also been suggested as a driver for reduced mortality in the long term. In a Swedish register-based analysis, lower mortality risk after surgery was seen in patients with diabetes and a weight reduction exceeding 2 BMI units^30^, in line with the observed weight reduction in patients from the usual care group who achieved remission in the current report. Moreover, in an exploratory analysis of long-term data from the Look AHEAD study, it was shown that the group of patients that achieved >10% 1-year weight loss after intensive lifestyle intervention lowered their mortality risk by more than 20% over 16 years of follow-up^33^. Although the studies mentioned above demonstrate a strong association between weight loss and the likelihood of diabetes remission, it should be noted that the current study was not designed to evaluate a cause–effect relationship. Instead, its aim was to assess future outcomes in patients who managed to achieve remission. However, if the survival benefit proves to be partly attributable to weight loss, this would imply promising outcomes for the recently developed potent weight loss medications^34,35^.

T2D is a common risk factor for CVD and cancer, the two most common causes of death in people with obesity^2,6,36^. In this study, 2-year diabetes remission was associated with reduced incidence of CVD as well as CVD-related death but not with cancer or cancer-related death. Moreover, remission was associated with a reduced risk of incident microvascular complications.

### Strengths and limitations

Major strengths of the SOS study are the very long follow-up and the prospective controlled study design, follow-up examinations to determine diabetes status, and the possibility of acquiring information from comprehensive national registers. Moreover, all participants in both treatment groups of the SOS study were eligible for surgery, while this information is not available in retrospective studies^11^. Several limitations are noted. First, the SOS study was initiated before repeated measurements were routinely used for the diagnosis of T2D; thus, diabetes diagnosis was based on measurements at a single time point and/or the use of diabetes medication. Still, a sensitivity analysis that included only patients where a T2D diagnosis could be verified generated similar results. Second, although mortality and T2D were pre-specified endpoints of the SOS study, it was not powered for subgroup-stratified analyses as was performed here. Third, patients in the SOS study displayed a short average diabetes duration, and the results may, therefore, not be applicable to cohorts with longer diabetes durations. Fourth, the present analyses were conditioned on persons surviving and participating in the 2-year follow-up health examination. Fifth, the large majority of individuals in the SOS study are of Swedish ancestry, and it is unclear if the findings are generalizable to populations of other ethnic origin.

## Conclusion

Patients who achieved short-term diabetes remission (2 years) had increased life expectancy and especially decreased risk of cardiovascular death in this post-hoc analysis of data from the SOS study. Future studies should confirm these findings and identify the key mechanisms involved in these associations.

## Ethical approval

Seven regional ethics review boards (Gothenburg, Lund, Linköping, Örebro, Karolinska Institute, Uppsala, Umeå) approved the study (reference numbers 184-90 and T508-17).

## Consent

Written or verbal informed consent was obtained from all participants.

## Source of funding

This project was supported by grants from the Swedish Research Council (2021-01496 and 2020-01303), the Swedish state under the agreement between the Swedish government and the county councils, the ALF-agreement (ALFGBG-965955, ALFGBG-965046, ALFGBG-966076), the Health & Medical Care Committee of the Region Västra Götaland (VGFOUREG-931560, VGFOUREG-941125), the Swedish Heart Lung Foundation (20210186), the Gothenburg Medical Society and the Adlerbert Research Foundation.

## Author contribution

L.M.S.C., M.P., B.C., and K.S.: designed the study; K.S.: wrote the paper with contributions from all authors; M.P.: designed and conducted the statistical analysis; L.M.S.C., K.S., P.J., and J.C.A.A.: organized register linkage with the Swedish authorities. All authors contributed to the acquisition and interpretation of data, critically reviewed the report, contributed to the revision, and approved the final version of the manuscript.

## Conflicts of interest disclosure

B.C. and C.K. are employed by AstraZeneca and hold stocks in the same company. No other conflicts of interest relevant to this study were reported.

## Research registration unique identifying number (UIN)

1. Name of the registry: ClinicalTrials.gov.
2. Unique identifying number or registration ID: NCT01479452.
3. Hyperlink to your specific registration (must be publicly accessible and will be checked): https://clinicaltrials.gov/study/NCT01479452

## Guarantor

L.M.S.C., M.P., and K.S. are the guarantors of this work and, as such, had full access to all the data in the study and take responsibility for the integrity of the data and the accuracy of the data analysis. K.S. had final responsibility for the decision to submit for publication.

## Data availability statement

The requested information is subject to legal restrictions according to national legislation. Confidentiality regarding personal information in studies is regulated in the Public Access to Information and Secrecy Act (SFS 2009:400), OSL. A request to get access to public documents can be rejected or granted with reservations by the University of Gothenburg. If the University of Gothenburg refuses to disclose the documents, the applicant is entitled to a written decision that can be appealed to the administrative court of appeal.

## Provenance and peer review

Not commissioned, externally peer-reviewed.

## Supplementary Material

SUPPLEMENTARY MATERIAL
