# Supplementary material for: Mortality in relation to diabetes remission in Swedish Obese Subjects – a prospective cohort study
Source: Int J Surg. 2024 Jun 19;110(10):6581–90. doi: 10.1097/JS9.0000000000001807 (PMC11487030; doi:10.1097/JS9.0000000000001807)
Supplement: SUPPLEMENTARY MATERIAL [file js9-110-6581-s001.docx]

Supplementary Appendix

Mortality in relation to diabetes remission in Swedish Obese Subjects -a prospective cohort study

by

Carlsson LMS, Carlsson B, Jacobson P, Karlsson C, Andersson-Assarsson JC, Kristensson FM, Ahlin S, Svensson P-A, Taube M, Näslund I, Karason K, Peltonen M, Sjöholm K.

| **Table of contents:** | **Page** |
| --- | --- |
| **SOS study design** | 2 |
| **sFigure 1.** Survival in remission and non-remission subgroups. | 3 |
| **sFigure 2.** Sensitivity analysis of overall mortality. | 4 |
| **sFigure 3.** Body mass index (A), absolute (B) and relative (C) changes in body mass index and absolute changes in body weight (D) over a period of 15 years. | 5 |
| **sTable 1.** International Classification of Diseases and intervention codes used for detection of diabetes-related morbidity | 6 |
| **sTable 2.** Baseline characteristics and 2-year changes in the full cohort by 2-year remission status | 8 |
| **sTable 3.** Hazard ratios with 95% confidence intervals (CI) for total mortality among those who achieved 2-year remission compared to those who did not, with complete case analysis and with multiple imputation of missing remission data. | 10 |
| **sTable 4.** Sub-hazard ratios (sub-HR) with 95% confidence intervals (CI) from Fine and Gray competing risk regression models for causes of death with ≥10 events. | 11 |

**SOS study design**

As a result of recruitment campaigns in the mass media and at primary health-care centers, 6905 persons completed a matching examination and 5335 of them were eligible for inclusion in the Swedish Obese Subjects (SOS) study. The per-protocol surgery group constituted n=2007 individuals electing surgery and a contemporaneously matched control group (n=2040) was created based on 18 matching variables. The 18 variables were sex, postmenopausal status, age, smoking status, diabetes, weight, height, hip circumference, waist circumference, systolic blood pressure, triglycerides, total cholesterol, current health, monotony avoidance, psychasthenia, quantity of social support, quality of social support and stressful life events. The matching was not performed at an individual level. Instead, the matching algorithm selected controls so that the current mean values of the 18 matching variables in the control group became as similar as possible to the current mean values in the surgery group according to the method of sequential treatment assignment (Pocock SJ, Simon R. Sequential treatment assignment with balancing for prognostic factors in the controlled clinical trial. Biometrics 1975; 31(1): 103-15).

The study began on the day of surgery for participants in the surgery group as well as for their matched usual care controls. Physical examinations were performed at baseline and after 0.5 and 1, 2, 3, 4, 6, 8, 10, 15 and 20 years. Urine and fasting blood samples obtained at baseline and after 2, 10, 15 and 20 years were analysed at the Central Laboratory, Sahlgrenska University Hospital, Gothenburg, Sweden, accredited according to International Organization for Standardization/International Electrochemical Commission 15189:2007 standards.The exclusion criteria were earlier surgery for gastric or duodenal ulcer, earlier bariatric surgery, gastric ulcer during the past 6 months, ongoing malignancy, active malignancy during the past 5 years, myocardial infarction during the past 6 months, bulimic eating pattern, drug, or alcohol abuse, psychiatric or cooperative problems contraindicating bariatric surgery, other contraindicating conditions (e.g. chronic glucocorticoid or anti-inflammatory treatment).

**sFigure 1.** Survival in remission and non-remission subgroups. Shown are the proportion surviving (opaque lines) and the estimate of survival from an unadjusted Gompertz regression model extrapolated up to 50 years (fainter lines).

**sFigure 2.** Sensitivity analysis of overall mortality in the full cohort (left panel), surgery (middle panel) and usual care (right panel) groups of the SOS study stratified by diabetes remission at 2 years. The analysis included only individuals where diabetes identified with a single measure of blood glucose or HbA1c could be verified by another measurement at the matching and/or baseline examination. Per-protocol analysis adjusted for age, sex, smoking, inclusion year, diabetes duration, previous CVD, hypertension and total cholesterol. IR/1000, incidence rate per 1,000 person-years.

**sFigure 3.** Body mass index (A), absolute (B) and relative (C) changes in body mass index and absolute changes in body weight (D) over a period of 15 years. Lines are estimated means from a mixed model with adjustment for sex and age.

## sTable 1. International Classification of Diseases and intervention codes used for detection of diabetes-related morbidity.

|  | **ICD-9** | **ICD-10** | **Procedure codes of the National Swedish Board of Health and Welfare** | |
| --- | --- | --- | --- | --- |
| **CARDIOVASCULAR^1^** |  |  |  |  |
| Centrally located macrovascular disease (myocardial infarction or stroke) | 410  431  433-434  436 | I21-I22  I61  I63-I66 |  |  |
| **MICROVASCULAR^2^** |  |  | Classification of operations‡ Ed. 5, 1985 & 6, 1989 (both including also non-surgical procedures) | Classification of surgical procedures (KKÅ) 1997, Temporary list of non-surgical procedures (TÅL) 1997. Swedish Classifications of Health Interventions (KVÅ) 2007^#^ including both surgical (KKÅ) and non-surgical (KMÅ) procedures. |
| Kidney complications | 250D  V42A  791A  584-586  V45B  V56A  V56W | E11.2  E10.2*  E14.2  Z940  N08.3  R80  N39.1  N17-N19  Z99.2  Z49 | 6070  6080-6081  9211-9214 | KAS00  KAS10  KAS20  KAB00-KAB01  DR015-DR016  DR020  V9211-V9212  V9507  DR023-DR024  JAK10  TJA20  TJA33  V9213-V9214  V9531-V9532 |
| Eye complications | 250E | E11.3  E10.3*  E14.3  H280  H36.0 | 1630-1638 | CKC-CKD |
| Neurological complications | 250F  357E | E11.4  E10.4*  E14.4  G73.0  G99.0  G59.0  G63.2 |  |  |

‡ First edition of Classification of Operations (Swedish: “Klassifikation av Operationer”) was printed by the National Swedish Board of Health and Welfare in 1963.

**#** KVÅ is available only online ([www.socialstyrelsen.se/statistik-och-data/klassifikationer-och-koder/kva](http://www.socialstyrelsen.se/statistik-och-data/klassifikationer-och-koder/kva)) and is updated annually since 2007. Older code lists were printed by the National Swedish Board of Health and Welfare.

* In the Swedish National Patient Registry and the Cause of Death Registry, complications of some typical type 2 diabetic individuals have erroneously been coded as type 1 diabetes (i.e. with E10# codes), particularly if they have obtained insulin treatment. We have therefore included both E11 (type 2) and E10 (type 1) codes in our searches for microvascular events.

**References**

1. Sjöström L, Peltonen M, Jacobson P, et al. Bariatric surgery and long-term cardiovascular events. JAMA 2012;307(1):56-65.

2. Carlsson LMS, Sjöholm K, Karlsson C, et al. Long-term incidence of microvascular disease after bariatric surgery or usual care in patients with obesity, stratified by baseline glycaemic status: a post-hoc analysis of participants from the Swedish Obese Subjects study. The lancet Diabetes & endocrinology 2017;5(4):271-279.

**sTable 2.** Baseline characteristics and 2-year changes in the full cohort by 2-year remission status

|  | **Full cohort** | | |
| --- | --- | --- | --- |
|  | **Remission** | **Non-remission** | **P value** |
| Body weight, kg |  |  |  |
| Baseline | 124.2±19.7 | 117.1±16.8 | <0.001 |
| 2 years | 96.7±18.7 | 109.6±18.4 | <0.001 |
| Absolute change | -27.4±17.1 | -7.5±11.1 | <0.001 |
| Waist circumference, cm |  |  |  |
| Baseline | 128.9±12.4 | 124.0±10.2 | <0.001 |
| 2 years | 108.1±14.1 | 118.8±12.0 | <0.001 |
| Absolute change | -20.8±13.6 | -5.3±9.7 | <0.001 |
| Body mass index, kg/m^2^ |  |  |  |
| Baseline | 42.7±5.0 | 40.2±4.4 | <0.001 |
| 2 years | 33.3±5.4 | 37.6±5.2 | <0.001 |
| Absolute change | -9.4±5.8 | -2.6±3.8 | <0.001 |
| Blood glucose, mmol/L |  |  |  |
| Baseline | 7.4±2.3 | 8.8±2.9 | <0.001 |
| 2 years | 4.4±0.6 | 8.2±2.9 | <0.001 |
| Absolute change | -3.0±2.3 | -0.6±3.4 | <0.001 |
| HbA1c, mmol/mol |  |  |  |
| Baseline | 56.0±14.9 | 65.4±16.0 | <0.001 |
| 2 years | 39.5±4.3 | 65.7±17.6 | <0.001 |
| Absolute change | -16.4±14.9 | 0.2±19.6 | <0.001 |
| Serum insulin, pmol/L |  |  |  |
| Baseline | 169.7±93.4 | 151.1±93.6 | 0.017 |
| 2 years | 73.1±41.1 | 126.2±85.0 | <0.001 |
| Absolute change | -96.5±89.6 | -25.3±90.1 | <0.001 |
| HOMA-IR |  |  |  |
| Baseline | 10.6±7.6 | 11.0±7.8 | 0.552 |
| 2 years | 2.7±1.7 | 8.7±6.5 | <0.001 |
| Absolute change | -7.9±7.4 | -2.3±8.4 | <0.001 |
| Total cholesterol, mmol/l |  |  |  |
| Baseline | 5.9±1.3 | 5.7±1.1 | 0.024 |
| 2 years | 5.7±1.2 | 5.6±1.2 | 0.118 |
| Absolute change | -0.2±1.0 | -0.2±0.9 | 0.322 |
| HDL cholesterol, mmol/l |  |  |  |
| Baseline | 1.2±0.3 | 1.2±0.3 | 0.905 |
| 2 years | 1.5±0.4 | 1.3±0.3 | <0.001 |
| Absolute change | 0.3±0.3 | 0.1±0.2 | <0.001 |
| Non-HDL cholesterol, mmol/l |  |  |  |
| Baseline | 4.6±1.2 | 4.4±1.2 | 0.211 |
| 2 years | 4.2±1.2 | 4.2±1.1 | 0.617 |
| Absolute change | -0.4±0.9 | -0.2±0.8 | 0.044 |
| LDL cholesterol, mmol/l |  |  |  |
| Baseline | 3.4±1.0 | 3.3±1.0 | 0.044 |
| 2 years | 3.4±1.0 | 3.1±0.9 | <0.001 |
| Absolute change | 0.0±0.8 | -0.1±0.7 | 0.071 |
| Triglycerides, mmol/l |  |  |  |
| Baseline | 2.8±2.4 | 2.7±1.8 | 0.609 |
| 2 years | 1.7±1.0 | 2.4±1.5 | <0.001 |
| Absolute change | -1.1±2.0 | -0.3±1.4 | <0.001 |
| Systolic blood pressure, mm Hg |  |  |  |
| Baseline | 148.9±19.2 | 147.0±19.0 | 0.252 |
| 2 years | 138.3±20.7 | 143.0±19.5 | 0.005 |
| Absolute change | -10.7±19.9 | -4.0±19.2 | <0.001 |
| Diastolic blood pressure, mm Hg |  |  |  |
| Baseline | 91.3±11.6 | 88.6±11.3 | 0.004 |
| 2 years | 83.9±11.0 | 85.4±10.2 | 0.094 |
| Absolute change | -7.4±11.4 | -3.2±10.9 | <0.001 |

Data are mean ± SD.

**sTable 3.** Hazard ratios with 95% confidence intervals for total mortality among those who achieved 2-year remission compared to those who did not, with complete case analysis and with multiple imputation of missing remission data.

|  | **Missing data on 2-year diabetes remission, %** | **Hazard ratio, adj.*** | **95% CI** | **p-value** |
| --- | --- | --- | --- | --- |
| **Complete case analysis (persons with unknown 2-year diabetes remission status excluded):** |  |  |  |  |
| Full | 13.6 | 0.71 | 0.54-0.95 | 0.019 |
| Surgery | 11.3 | 0.80 | 0.51-1.25 | 0.330 |
| Usual care | 16.5 | 0.68 | 0.40-1.18 | 0.171 |
|  |  |  |  |  |
| **Imputed analysis^§^ (persons with missing 2-year diabetes remission status included with predicted data)** |  |  |  |  |
| Full | “0” | 0.72 | 0.55-0.94 | 0.015 |
| Surgery | “0” | 0.83 | 0.55-1.25 | 0.375 |
| Usual care | “0” | 0.76 | 0.45-1.28 | 0.300 |

* Hazard ratios are adjusted for sex and baseline age, body mass index, smoking status, inclusion year, diabetes duration, previous CVD, hypertension and total cholesterol.

**^§^** The missing 2-year diabetes status (remission/non-remission) data were imputed, using the following baseline variables as predictors for the missing diabetes status: age, sex, smoking, treatment group (surgery/control), body mass index at baseline, and inclusion year. The number of imputations (number of complete datasets with missing values imputed) were set to 30.

**sTable 4.** Sub-hazard ratios (sub-HR) with 95% confidence intervals (CI) from Fine and Gray competing risk regression models for causes of death with ≥10 events.

|  | **Adj. sub-HR (95% CI)**^1^ | **Adj. p for sub-HR**^1^ |
| --- | --- | --- |
| Cardiac | 0.51 (0.31-0.83) | 0.007 |
| MI | 0.43 (0.19-0.97) | 0.042 |
| Heart failure | 0.51 (0.16-1.67) | 0.267 |
| Sudden death | 0.63 (0.31-1.28) | 0.201 |
| Stroke | 0.78 (0.26-2.31) | 0.656 |
| Infection | 0.35 (0.14-0.88) | 0.026 |
| Causes other than disease | 2.41 (0.60-9.61) | 0.212 |
| Other or multiple conditions | 0.47 (0.11-2.07) | 0.320 |

^1^Adjusted for sex, age, BMI, smoking, year of inclusion, diabetes duration, previous CVD, hypertension and total cholesterol.
